# Monitoring Cyclosporine Absorption: A Step Beyond Therapeutic Drug Monitoring

**DOI:** 10.5812/numonthly.7463

**Published:** 2012-12-15

**Authors:** Po-Chang Lee

**Affiliations:** 1 National Cheng Kung University Hospital, Taipei, Taiwan

**Keywords:** Cyclosporine, Drug Monitoring

Dear Editor,

In their recent large, retrospective, cross-sectional study with 7702 renal-transplant patients, Einollahi et al. showed that cyclosporine (CsA) absorption (CA) steadily increases over 3 years post transplant (1). They also indicated that despite the day-to-day and inter-individual variability in CA, the CsA levels are routinely monitored by measuring the predose blood trough level (C0) or the 2-h post-dose level (C2). Their approach was to use the C2/C0 ratio as a measure of CA because it takes into account both the elimination and absorption phases, which are required to identify high and low CsA absorbers (1).

According to the convention, the therapeutic target ranges for C0 and C2 of CsA were higher during the first 3 months post-transplant. Although the CsA dose was gradually reduced thereafter, as evidenced by the decreases in C0 and C2, to avoid CsA nephrotoxicity, CA increased during the 3-year post-transplant period. This increase in CA had a significant correlation with allograft function. CA levels also correlated with lower recipient age, hypokalemia, hypernatremia, hypotriglyceridemia, anemia, higher creatinine concentration, and lower LDL levels (1). 

Literature suggests that increase in CA after renal transplantation is influenced by numerous factors modifying CsA pharmacokinetics, including food, concomitant medication, CsA nephropathy, and decrease in CYP3A4 and P-glycoprotein levels (1). Increased serum creatinine and triglyceride levels, and hypokalemia are also associated with increased CsA levels (1, 2).

Einollahi et al., through statistical analysis, showed that many of these factors correlated with higher CA. However, an important noteworthy point is that this study included long-term transplant patients, and the changes in CsA dose at the 4 time points post transplant were associated with decreased C0 and C2 levels and increased CA over a 3-year period (1). This finding suggests that the observed increase in CA is perhaps CsA specific, and highlights the role of inter-individual variability in CsA absorption as an important factor that must be considered for preventing CsA overuse-induced nephrotoxicity. Further, CsA co-administration increases the levels of other immunosuppressants such as mycophenolic acid, showing formulation-based variations (3). Therefore, further studies are needed to clarify the effect of CA on concomitant medications. 

Chapman et al. discussed the causes of chronic allograft nephropathy (CAN) and concluded that late identification of CAN leading to graft loss is an indication that the intervening strategies tend to be “too little and far too late” (4). A review by Vanrenterghem suggested that an individualized regimen requires selection of an immunosuppressive protocol and monitoring of individual drug-related toxicity, risk factors, and donor organ characteristics (5). Regarding toxicity, C0 does not indicate the amount of absorption, whereas C2 lacks the trough level aspect. Therefore, in renal-transplant patients on CsA, the C2/C0 ratio is a better marker and evidence of increasing CA as it indicates a fold increase in CsA levels from trough levels. In keeping with the spirit of doing “a little more” in patient interest, monitoring the C2/C0 ratio can serve as a balanced and individualized intervention strategy, which can also be applied to other drugs with a narrow therapeutic index.
